# The impact of peri-natal stress on psychosis risk: results from the Bo-FEP incidence study

**DOI:** 10.1186/s13104-020-04992-9

**Published:** 2020-03-16

**Authors:** Tiziano De Matteis, Giuseppe D’Andrea, Jatin Lal, Domenico Berardi, Ilaria Tarricone

**Affiliations:** 1 Bologna Transcultural Psychosomatic Team (BoTPT), Department of Medical and Surgical Sciences, Alma Mater Studiorum Bologna University, Bologna, Italy; 2 Department of Biomedical and Neuromotor Sciences, Alma Mater Studiorum, Bologna University, Via Giovanni Masserenti, 9 – Pavillon 11, 40138 Bologna, Italy; 3 Department of Medical and Surgical Sciences (DIMEC), University of Bologna; Clinica Medica, Policlinico Sant’Orsola-Malpighi, Via Masserenti 9, 40138 Bologna, Italy

**Keywords:** First-episode psychosis, Peri-natal stress, Obstetric complications, Risk of psychosis

## Abstract

**Objective:**

According to the gene-environment interaction model the pathogenesis of psychosis relies on an adverse neuro-socio-developmental pathway. Perinatal stress represents an important risk factor for the development of psychosis because of the increasingly evident interference with socio-neuro-development in the earlier phases of life. We aim to investigate the correlation of perinatal risk factors with the onset of psychosis with a case–control–incidence study.

**Results:**

Patients (and their mothers) were eligible if they presented with first-episode psychosis at the Bologna West Community Mental Health Centre (Bo-West CMHC) between 2002 and 2012. The Bo-West CMHC serves a catchment area of about 200,000 people. The controls were recruited in the same catchment area and study period. 42 patients, 26 controls and their mothers were included. We collected the history of peri-natal stress and calculated crude and adjusted Odds Ratios for onset of first-episode psychosis. Adjusted logistic regression showed that psychosis onset was significantly associated with stressful situations during pregnancy, lower level of maternal physical health before or during pregnancy, use of anti-inflammatory drugs during pregnancy, and low level of maternal education. The results of our study suggest that stress during perinatal period increases the risk of developing psychosis.

## Introduction

The pathogenesis of psychosis relies on several causal factors according to the gene-environment (GxE) interaction model [1–4]. Prenatal and perinatal complications represent important risk factors for the development of psychosis because they interfere with neurodevelopment [5].

Pre- and perinatal risk factors of psychosis in the offspring can be grouped as pregnancy-dependent or pregnancy-independent. Several of these variables, including the exposure to stressful or traumatic events during pregnancy [6–9], low socio-economic status (SES) [10, 11], and low level of maternal education [12], have been correlated with higher risk of psychotic onset in the offspring. Disadvantaged social conditions may be associated with a higher number of obstetric complications (OCs), more stressful life events, specific (tuberculosis and sexually transmitted diseases) and non-specific infections [13], potentially risky behaviors such as poor medical monitoring of pregnancy and alcohol and tobacco consumption, food deficiencies [14, 15].

Several studies have explored the effect of maternal diseases on psychosis onset in offspring, such as maternal influenza (especially in the first trimester of pregnancy) [16], infections from Rubella Virus, Toxoplasma gondii [17] and HSV type 2 [18]. Increased risk of psychosis in the offspring was also correlated with maternal inflammation markers, such as elevated levels of interleukin-8 and C-reactive protein [13, 19, 20]. Additionally, birth in late winter and early spring constitute a risk factor for psychosis [21–23].

An increased risk of schizophrenia in offspring is associated with the mother’s being very young or older than 34 years [24]. Likewise, the father’s being older than 35 is associated with an increased risk of schizophrenia in offspring [25–27].

Epidemiological studies showed that OCs are associated with a double risk of schizophrenia in offspring [28, 29]. The main etiopathogenetic mechanism involved would seem to be hypoxia. [7, 10, 30].

Few studies have investigated simultaneously the effects of pregnancy-dependent vs pregnancy-independent risk factors on psychosis developing among offspring. The objective of our study is to analyze the prevalence and correlation with psychotic onset of pregnancy dependent and independent pre-, perinatal risk factors.

## Main text

### Methods

#### Recruitment

Patients between 18 and 64 years old with a first-episode psychosis (FEP) were identified among first accesses to the three CMHCs within tightly defined catchment areas in West–Bologna, Italy from January 2002 to December 2012 [31]. The inclusion criteria were based on those used in the World Health Organization (WHO) study [32]: i.e., presence of hallucinations, delusions, thought disorder, bizarre or disturbed behavior, negative symptoms, mania, or clinical suspicion of psychosis; absence of an organic cause or profound learning disability; and no previous contact with psychiatric services for psychotic symptoms. Case-notes were used to complete the Item Group Checklist (IGC), part of the Schedule for Clinical Assessment of Neuropsychiatry, Version 2.1 (SCAN; WHO, 1998), to collect symptom-related data at the time of presentation and 1 month later to ensure that cases met ICD-10 criteria for psychotic disorders [31].

Controls were healthy individuals and their mothers and were recruited from the same catchment area. To select a population-based sample of controls broadly representative of local populations in relation to age, gender, and ethnicity, a mixture of random and quota sampling was used. Individuals who agreed to take part were screened for a history of psychosis and were included only when they did not report past or current psychotic disorder [33].

Potential participants and their mothers were contacted after a preliminary opinion from the clinical psychiatrist in charge to evaluate whether they should be proposed for inclusion in the study. Interviews were also conducted on controls’ mothers. The interview was conducted by a trained mental health operator.

#### Measures

Subjects’ mothers were interviewed using the “Mother Interview”, formulated within the Genetic and Psychosis project (GAP) [34]. This consists of three basic sections: Section A-Demographic Survey (collects socio-demographic information about family members of the subject, especially at the time of pregnancy and birth); Section B-Questionnaire on Obstetric Complications (Pathological and obstetrical history of the mother; Pregnancy Complications; Delivery Complications; Neonatal Complications); Section C-First stages of development (any problems in early childhood). In order to highlight the presence of OCs in the history of each case or control under examination, the Lewis-Murray Scale [30] was used on the data obtained from the interview with the mother.

The sociodemographic information on the subjects was collected through the Medical Research Council (MRC) socio-demographic schedule [35], which gathers sociodemographic data as well as information relating to substance use and migratory history. The subjects were also evaluated via the Cannabis Experience Questionnaire (CEQ) [33, 36] and the Family Interview for Genetic Studies (FIGS) [37].

#### Statistical analyses

The group comparison methods used include the Chi square test and Fischer’s Exact test for categorical variables and one-way analysis of variance for continuous dependent variables. Logistic regression models were used to analyze associations between independent and dependent variables, as well as to estimate the odds ratio (OR)-when the data distribution made it possible- and confidence intervals at 95% (CI). Subsequently adjustments were made for confounding factors (age and sex) with multivariate logistic regression. All the statistical analyses were conducted with SPSS for Windows 23.0

### Results

#### Forty-two patients, 26 controls, and their mothers were included (Table 1)

250 cases were initially recruited, but it was possible to interview the mothers in 42 cases (16.8%). In 208 cases no interview was possible because either the patients were no longer in contact with the service; their mothers were deceased, they were adopted, the mothers were abroad, clinicians did not recommend the interview, or patients and/or their mothers did not consent. The cases included were significantly younger than those not included (28.05 vs 32.65 p = 0.006) with no other difference between the two groups.

**Table 1:** Socio-demographics characteristics

	Cases	Controls
n (%)	42 (100)	26 (100)
Gender, n (%)		
Men **	26 (61.9)	9 (34.6)
Women **	16 (38.1)	17 (65.4)
Mean age of the participants (SD)	28.07 (8.52)	30.85 (5.98)
Origin, n (%)		
Natives	38 (90.5)	26 (100)
Emilia Romagna	28 (66.5)	11 (42.3)
Other regions	10 (23.8)	15 (57.7)
Migrants	4 (9.5)	0 (0.0)
Ethnicity, n (%)		
Caucasian	40 (95.2)	26 (100)
African	2 (4.8)	0 (0.0)
Social class (defined by the paternal working status at birth), generic, n (%)	39 (100)	25 (100)
Intermediate-high **	22 (56.4)	20 (80.0)
Low**	17 (43.6)	5 (20.0)
Social class (defined by the paternal working status at birth), specific, n (%)		
High-level managerial and professional activities	8 (20.5)	9 (36.0)
Low-level managerial and professional activity	2 (5.1)	2 (8.0)
Intermediate occupation	5 (12.8)	4 (16.0)
Employees/own small business	5 (12.8)	3 (12.0)
Low-level technical activities	2 (5.1)	2 (8.0)
Semi-routine occupations	4 (10.3)	0 (0.0)
Routine occupations	12 (30.8)	5 (20.0)
Unemployed	1 (2.6)	0 (0.0)
Family history of psychiatric diseases (1st degree relatives), n (%)	31 (100)	20 (100)
Yes	8 (25.8)	7 (35.0)
Family history of psychosis	1 (3.4)	0 (0.0)

*p < 0.1 e > 0.05; **p < 0.05 e > 0.001; ***p < o = 0.001

Sociodemographic characteristics of cases and controls were reported in Table 1. Cases were more frequently men (26, 61.9%) than controls (9, 34.6%, c sq = 4.79, p = 0.029), The mean age was 28.07 years (+ 8.52) at the time of onset. Greater prevalence of high and intermediate social class levels was found among controls (80% vs 56.4%, c sq = 3.75, p = 0.053). There were no other statistically significant differences among cases and controls.

Table 2 reports characteristics of mothers of cases and controls and frequency of the obstetric complications. The mean age of mothers at delivery was 26.88 years for cases and 28.46 years in controls. The mean paternal age was 31.08 for cases, and 31.81 for controls. Mothers were mostly married, working, homeowners, living with their partner and/or children. A more frequent history of medical disorders and use of drugs (12, 34.3% vs 3, 12.5%), particularly anti-inflammatory drugs (7, 20.6% vs 0, 0.0%), were more frequently found among mothers of cases compared with mothers of controls. Moreover, mother of cases (24, 57.1%) reported more frequently negative memories during pregnancy compared to mothers of controls (2, 7.7%). No other difference was found between mother of cases and mothers of controls.

**Table 2:** Association between obstetric complications and first-episode psychosis

Health status of the mothers	Cases	Controls
n (%)	42 (100.0)	26 (100.0)
First child	29 (69.0)	15 (57.7)
Second child	8 (19.0)	8 (30.8)
Third or further child	5 (11.9)	3 (11.5)
Mean age of the parents at cases’ births (SD)		
Mothers’ age	26.88 (5.4)	28.46 (5.3)
Fathers’ age	31.08 (6.06)	31.81 (5.7)
Mean age of the mothers at the interview (SD)	58.57 (9.5)	58.96 (5.4)
Maternal disorders history, n (%)	42 (100.0)	26 (100.0)
Negative***	20 (46.6)	24 (92.3)
Positive***	22 (52.4)	2 (7.7)
Endocrine-Metabolic disorders	5 (11.9)	0 (.0)
Cardio-vascular disorders	4 (9.5)	0 (0.0)
Respiratory disorders	2 (4.8)	0 (0.0)
Neurological disorders	1 (2.4)	0 (0.0)
Gynaecological disorders *	9 (21.4)	1 (3.8)
Other	9 (21.4)	2 (7.7)
Pathological obstetric history, n (%)	42 (100.0)	26 (100.0)
Not applicable	29 (69.0)	14 (53.8)
Negative	10 (23.8)	10 (38.5)
Positive	3 (7.1)	2 (7.7)
OCs, n (%)	42 (100.0)	26 (100.0)
Present	27 (65.9)	20 (76.9)
Absent	14(34.1)	6 (23.1)
Negatives memories (e.g. trauma), n (%)	42 (100.0)	26 (100.0)
No***	18 (42.9)	24 (92.3)
Yes***	24 (57.1)	2 (7.7)
Use of drugs-alcohol-substances in pregnancy		
Not-users	24 (57.1)	14 (53.8)
Users	18 (42.9)	12 (46.2)
Alcohol during pregnancy, n (%)	15 (35.7)	11 (42.2)
Monthly or less	3 (7.1)	1 (3.8)
2–3 time/month	5 (11.9)	1 (3.8)
2–3 times/week	5 (11.9	8 (30.8)
Every day	2 (4.8)	1 (3.8)
Smoking during pregnancy, n (%)	7 (15.8)	3 (11.5)
6–9 sigarettes/die	6 (14.3)	2 (7.7)
10–20 sigarettes/die	1 (2.4)	1 (3.8)
Other smokers at home	18 (43.9)	14 (56.0)
Other Substances during pregnancy, n (%)	0 (0.0)	0 (0.0)
Medicinal drugs during pregnancy, n (%)	35 (100.0)	24 (100.0)
Yes*	12 (34.3)	3 (12.5)
No*	23 (65.7)	21 (87.5)
Anti-inflammatory drugs**	7 (20.6)	0 (0.0)

*p < 0.1 e > 0.05; **p < 0.05 e > 0.001; ***p < o = 0.001

The following associations were found to be significant by logistic regression analysis adjusted for sex and age:

- highly stressful situations during pregnancy (c sq = 16.62, p = 0.000), OR = 16.0 (95% CI 3.3–76.6; p = 0.001); the risk remains significant even when adjusted for age and gender in multivariate logistic regression analysis (OR = 23.8, 95% CI 4.2–134.2, p = 0.000);
- a lower level of maternal physical health before or during pregnancy (c sq = 16.62, p = 0.000), OR = 16 (95% CI 3.3–76.6; p = 0.001); the risk remains significant even when adjusted for age and gender in multivariate logistic regression analysis (OR = 16.8, 95% CI 3.3–86.0,p = 0.001).
- use of anti-inflammatory drugs during pregnancy (c sq = 5.61; p = 0.018).
- low level of maternal education (c sq = 5.49; p = 0.019), OR = 4.1(95% CI 1.2–14.2; p = 0.024); the risk remains significant even when adjusted for age and gender in multivariate logistic regression analysis (OR = 5.08, 95% CI 1.3–20.5, p = 0.023).

### Experience of OCs prior to pregnancy was rare without significant differences between cases and controls

The use alcohol during pregnancy was no different between the groups and there wasn’t any difference in cigarette consumption during pregnancy between cases and controls. Around half of the fathers smoked at home during pregnancy without any statistically difference. No mother admitted to having used any substance during pregnancy.

Most mothers nursed, in similar proportions between cases and controls. Patients’ mothers breastfed for longer, for a mean of 6.85 months compared to 4.1 months for controls (t test = 2.083, p = 0.041). Alcohol was the most used substance (5, 14.3% of cases, 4, 17.4% of controls), followed by smoking (3, 8.1% in cases; 1, 4.3% in controls) and medication (3, 8.8% in cases; 2, 8.7% in controls). The use of potentially risky substances (alcohol, medications and drugs) was not significantly different between cases and controls.

Infections during pregnancy were reported by 3 mothers (11.5%) of controls and 2 (4.9%) of patients. For both cases and controls, urogenital infection and influenza were the most common.

An excessive weight gain was found in 6 (14.6%) mothers of patients and 3 (11.5%) of controls.

There was a slightly higher incidence of OCs in mothers of patients than in those of controls without any statistically significant difference (14, 34.1%, vs 6, 23.1%; c sq = 0.931, p = 0.335). Eight out of 14 OCs (19%) among cases and 4 (15%) out of 6 among controls occurred during pregnancy and consisted of a threat of abortion or ante-partum hemorrhage. OCs during delivery and postnatal period numbered 11 in cases and 7 in controls.

## Discussion

Our study showed that pre-natal—independent of pregnancy—risk factors, related to stress-trauma and poor maternal health conditions during pregnancy are associated with psychosis onset in offspring. In addition, we found a lower level of education in mothers of offspring with FEP.

One possible mediator between adverse environmental conditions during pregnancy and psychosis development in offspring may be the high level of stress experienced in pregnancy, as reported by mothers of our patients, with an age and gender adjusted OR of 24. Examples of stress factors reported by mothers in the Mother Interview in our study are relational problems in family life, major health problems, need to work hard or in unhealthy environments. Stress during pregnancy correlates with changes in the hypothalamus-pituitary axis that regulates cortisol secretion in the offspring. Maternal glucocorticoids seem to have a great effect on the child’s stress with dysregulation in the dopaminergic system and a depression of its axis would lead to negative symptoms and cognitive symptoms [38]. In addition, there may be direct action on the expression of NMDA receptors, which are reduced in the hippocampus of patients undergoing prenatal stress and could predispose to greater stress vulnerability in later periods of perinatal development [4, 39].

Our finding of a greater use of anti-inflammatory drugs by mothers of patients during pregnancy, especially acetylsalicylic acid, is consistent with the evidence that their use during pregnancy correlates with neuro-development disorders. Its action leads to a deficiency of prostaglandin and essential fatty acids (constituents of Phospholipid membranes) which could alter the membrane structure in fetal brain development [40].

We would have expected a significant difference between cases and controls in the frequency of possible pregnancy-related risk factors such as OCs evaluated by the Lewis Murray Scale. As evidenced by previous studies, such events are approximately twice as frequent in those suffering from psychosis than in the general population [28]. It should be noted that OCs are rare events, which need to be better studied in larger samples. According to the Italian Certificate of Birth Assistance data of 2009, only 0.8% of newborns, within 5 min of birth, report an Apgar index of < 7 indicative of severe depression and therefore neonatal suffering and high mortality risk, which corresponds to approximately 1% of babies born weighing < 1500 g.

## Conclusions

Despite the limitations, this study offers interesting results, especially regarding the role of pre-natal independent-of-pregnancy risk factors in the development of psychosis. Of interest are the correlations between psychosis and:

1. Poor health during pregnancy, including the use of analgesics and anti-inflammatory drugs in pregnancy and impaired physical health of the mother at the time of conception;
2. Exposure to stressful or traumatic events.

The results of our study suggest that from the pre-natal phase onward attention should be given on avoiding stress and preventing its adverse effects on mother and child health, i.e. acting early in the gene-environment interaction. The role of genetic vulnerability will be better illustrated when the results of the EUGEI study are published/discussed. We therefore believe that potential risk factors before and during pregnancy, and not just OCs, should be studied and a preventive/early intervention strategy should be implemented. A more in-depth study of these risk factors and a more multidisciplinary approach to pregnancy-care could lead to primary prevention interventions targeting psychosis, such as raising the awareness of mothers and their social and familiar context about the harmful effect of exposure to stress.

## Limitations

- The number of participants is relatively small;
- The two groups differed in gender distribution, with men being more represented in the case group and women in the control group;
- The nature of the interviews and retrospective investigation made the study vulnerable to recall bias.

## Data Availability

The data supporting the findings of this study are available from the corresponding author on reasonable request.

## Abbreviations

SES: Socio-economic status
OCs: Obstetric complications
HSV: Herpes simplex virus
FEP: First-episode psychosis
CMHC: Community mental health centre
ICD: International classification of diseases
WHO: World Health Organization
GAP: Genetic and psychosis project
MRC: Medical Research Council
CEQ: Cannabis Experience Questionnaire
FIGS: Family Interview for Genetic Studies
OR: Odds ratio
CI: Confidence interval
c sq: Chi square

## References

[1] Uher R (2014). Gene-environment interactions in severe mental illness. Front Psychiatry..

[2] Van Os J, Kapur S (2012). Schizophrenia. Lancet.

[3] Mäki P, Riekki T, Miettunen J, Isohanni M, Jones PB, Murray GK (2010). Schizophrenia in the offspring of antenatally depressed mothers in the northern Finland 1966 birth cohort: relationship to family history of psychosis. Am J Psychiatry.

[4] Osborne S, Biaggi A, Chua TE, Du Preez A, Hazelgrove K, Nikkheslat N (2018). Antenatal depression programs cortisol stress reactivity in offspring through increased maternal inflammation and cortisol in pregnancy: the psychiatry research and motherhood–depression (PRAM-D) Study. Psychoneuroendocrinology..

[5] McCoy BM, Rickert ME, Class QA, Larsson H, Lichtenstein P, D’Onofrio BM (2014). Mediators of the association between parental severe mental illness and offspring neurodevelopmental problems. Ann Epidemiol.

[6] Huttunen MO, Niskanen P (1978). Prenatal loss of father and psychiatric disorders. Arch Gen Psychiatry.

[7] Khashan AS, Abel KM, McNamee R, Pedersen MG, Webb RT, Baker PN (2008). Higher risk of offspring schizophrenia following antenatal maternal exposure to severe adverse life events. Arch Gen Psychiatry.

[8] Van Os J, Selten JP (1998). Prenatal exposure to maternal stress and subsequent schizophrenia. The May 1940 invasion of The Netherlands. Br J Psychiatry..

[9] Fineberg AM, Ellman LM, Schaefer CA, Maxwell SD, Shen L, Chaudhury NH (2016). Fetal exposure to maternal stress and risk for schizophrenia spectrum disorders among offspring: differential influences of fetal sex. Psychiatry Res.

[10] Cannon M, Jones PB, Murray RM (2002). Obstetric complications and schizophrenia: historical and meta-analytic review. Am J Psychiatry.

[11] Werner S, Malaspina D, Rabinowitz J (2007). Socioeconomic status at birth is associated with risk of schizophrenia: population-based multilevel study. Schizophr Bull.

[12] Corcoran C, Perrin M, Harlap S, Deutsch L, Fennig S, Manor O (2009). Effect of socioeconomic status and parents’ education at birth on risk of schizophrenia in offspring. Soc Psychiatry Psychiatr Epidemiol.

[13] Brown AS, Hooton J, Schaefer CA, Zhang H, Petkova E, Babulas V (2004). Elevated maternal interleukin-8 levels and risk of schizophrenia in adult offspring. Am J Psychiatry.

[14] Eaton WW, Mortensen PB, Frydenberg M (2000). Obstetric factors, urbanization and psychosis. Schizophr Res.

[15] Webb RT, Wicks S, Dalman C, Pickles AR, Appleby L, Mortensen PB (2010). Influence of environmental factors in higher risk of sudden infant death syndrome linked with parental mental illness. Arch Gen Psychiatry.

[16] Brown AS, Begg MD, Gravenstein S, Schaefer CA, Wyatt RJ, Bresnahan M (2004). Serologic evidence of prenatal influenza in the etiology of schizophrenia. Arch Gen Psychiatry.

[17] Sørensen HJ, Mortensen EL, Reinisch JM, Mednick SA (2009). Association between prenatal exposure to bacterial infection and risk of Schizophrenia. Schizophr Bull.

[18] Mortensen PB, Pedersen CB, Hougaard DM, Nørgaard-Petersen B, Mors O, Børglum AD (2010). A Danish National Birth Cohort study of maternal HSV-2 antibodies as a risk factor for schizophrenia in their offspring. Schizophr Res.

[19] Canetta S, Sourander A, Surcel H-M, Hinkka-Yli-Salomäki S, Leiviskä J, Kellendonk C (2014). Elevated maternal C-reactive protein and increased risk of schizophrenia in a national birth cohort. Am J Psychiatry.

[20] Brown AS, Derkits EJ (2010). Prenatal infection and schizophrenia: a review of epidemiologic and translational studies. Am J Psychiatry.

[21] Córdova-Palomera A, Alemany S, Falcón C, Bargalló N, Goldberg X, Crespo-Facorro B (2014). Cortical thickness correlates of psychotic experiences: examining the effect of season of birth using a genetically informative design. J Psychiatr Res.

[22] Davies G, Welham J, Chant D, Torrey EF, McGrath J (2003). A systematic review and meta-analysis of Northern Hemisphere season of birth studies in schizophrenia. Schizophr Bull.

[23] Aschauer HN, Meszaros K, Willinger U, Reiter E, Heiden AM, Lenzinger E (1994). The season of birth of schizophrenics and schizoaffectives. Psychopathology.

[24] Cantor-Graae E, McNeil TF, Sjöström K, Nordström LG, Rosenlund T (1997). Maternal demographic correlates of increased history of obstetric complications in schizophrenia. J Psychiatr Res.

[25] Malaspina D, Harlap S, Fennig S, Heiman D, Nahon D, Feldman D (2001). Advancing paternal age and the risk of schizophrenia. Arch Gen Psychiatry.

[26] El-Saadi O, Pedersen CB, McNeil TF, Saha S, Welham J, O’Callaghan E (2004). Paternal and maternal age as risk factors for psychosis: findings from Denmark, Sweden and Australia. Schizophr Res..

[27] Lopez-Castroman J, Gómez DD, Belloso JJC, Fernandez-Navarro P, Perez-Rodriguez MM, Villamor IB (2010). Differences in maternal and paternal age between schizophrenia and other psychiatric disorders. Schizophr Res.

[28] Geddes JR, Verdoux H, Takei N, Lawrie SM, Bovet P, Eagles JM (1999). Schizophrenia and complications of pregnancy and labor: an individual patient data meta-analysis. Schizophr Bull.

[29] Hultman CM, Sparén P, Takei N, Murray RM, Cnattingius S, Geddes J (1999). Prenatal and perinatal risk factors for schizophrenia, affective psychosis, and reactive psychosis of early onset: case-control studyPrenatal and perinatal risk factors for early onset schizophrenia, affective psychosis, and reactive psychosis. BMJ.

[30] Lewis S, Owen M, York RM-N, Tamminga CA, Schulz SC (1989). Obstetric complications and schizophrenia: Methodology and mechanisms. Schizophrenia-A Scientific Focus.

[31] Tarricone I, Mimmi S, Paparelli A, Rossi E, Mori E, Panigada S (2012). First-episode psychosis at the West Bologna Community Mental Health Centre: results of an 8-year prospective study. Psychol Med.

[32] Jablensky A, Sartorius N, Ernberg G, Anker M, Korten A, Cooper JE (1992). Schizophrenia: manifestations, incidence and course in different cultures. A World Health Organization ten-country study. Psychol Med Monogr Suppl.

[33] Gayer-Anderson C, Jongsma HE, Di Forti M, Quattrone D, Velthorst E, de Haan L, Selten JP, Szöke A, Llorca PM, Tortelli A, Arango C, Bobes J, Bernardo M, Sanjuán J, Santos JL, Arrojo M, Parellada M, Tarricone I, Berardi D, Ruggeri M, Lasalvia A, Ferraro L, La Cascia C, La Barbera D, Menezes PR, Del-Ben CM; EU-GEI WP2 Group, Rutten BP, van Os J, Jones PB, Murray RM, Kirkbride JB, Morgan C. The European network of national schizophrenia networks studying gene-environment interactions (EU-GEI): incidence and first-episode case-control programme. Soc Psychiatry Psychiatr Epidemiol. 2020.10.1007/s00127-020-01831-x. (**Epub ahead of print**). 10.1007/s00127-020-01831-x 31974809

[34] Di Forti M, Morgan C, Dazzan P, Pariante C, Mondelli V, Marques TR (2009). High-potency cannabis and the risk of psychosis. Br J Psychiatry.

[35] van Os J, Rutten BP, Myin-Germeys I, Delespaul P, Viechtbauer W, European Network of National Networks studying Gene-Environment Interactions in Schizophrenia (EU-GEI) (2014). Identifying gene-environment interactions in schizophrenia: contemporary challenges for integrated, large-scale investigations. Schizophr Bull..

[36] Barkus EJ, Stirling J, Hopkins RS, Lewis S (2006). Cannabis-induced psychosis-like experiences are associated with high schizotypy. Psychopathology.

[37] Maxwell ME. Family Interview for Genetic Studies (FIGS): A Manual for FIGS. 1992.

[38] Malaspina D, Corcoran C, Kleinhaus KR, Perrin MC, Fennig S, Nahon D (2008). Acute maternal stress in pregnancy and schizophrenia in offspring: a cohort prospective study. BMC Psychiatry..

[39] Gi HS, Geum D, Chung S, Eun JK, Jo JH, Kim CM (2006). Maternal stress produces learning deficits associated with impairment of NMDA receptor-mediated synaptic plasticity. J Neurosci.

[40] Gunawardana L, Zammit S, Lewis G, Gunnell D, Hollis C, Wolke D (2011). Examining the association between maternal analgesic use during pregnancy and risk of psychotic symptoms during adolescence. Schizophr Res.

